# Biofunctionalized Structure and Ingredient Mimicking Scaffolds Achieving Recruitment and Chondrogenesis for Staged Cartilage Regeneration

**DOI:** 10.3389/fcell.2021.655440

**Published:** 2021-03-25

**Authors:** Zhen Yang, Hao Li, Yue Tian, Liwei Fu, Cangjian Gao, Tianyuan Zhao, Fuyang Cao, Zhiyao Liao, Zhiguo Yuan, Shuyun Liu, Quanyi Guo

**Affiliations:** ^1^Institute of Orthopedics, The First Medical Center, Chinese PLA General Hospital, Beijing Key Lab of Regenerative Medicine in Orthopedics, Key Laboratory of Musculoskeletal Trauma & War Injuries PLA, Beijing, China; ^2^School of Medicine, Nankai University, Tianjin, China; ^3^Department of Orthopedics, The First Affiliated Hospital of Zhengzhou University, Zhengzhou, China; ^4^Department of Bone and Joint Surgery, Renji Hospital, School of Medicine, Shanghai Jiao Tong University, Shanghai, China

**Keywords:** demineralized cancellous bone, extracellular matrix, transforming growth factor-β3, cell recruitment, pro-chondrogenesis, cartilage regeneration

## Abstract

It remains scientifically challenging to regenerate injured cartilage in orthopedics. Recently, an endogenous cell recruitment strategy based on a combination of acellular scaffolds and chemoattractants to specifically and effectively recruit host cells and promote chondrogenic differentiation has brought new hope for *in situ* articular cartilage regeneration. In this study, a transforming growth factor-β3 (TGF-β3)-loaded biomimetic natural scaffold based on demineralized cancellous bone (DCB) and acellular cartilage extracellular matrix (ECM) was developed and found to improve chondral repair by enhancing cell migration and chondrogenesis. The DCB/ECM scaffold has porous microstructures (pore size: 67.76 ± 8.95 μm; porosity: 71.04 ± 1.62%), allowing the prolonged release of TGF-β3 (up to 50% after 42 days *in vitro*) and infrapatellar fat pad adipose-derived stem cells (IPFSCs) that maintain high cell viability (>96%) and favorable cell distribution and phenotype after seeding onto the DCB/ECM scaffold. The DCB/ECM scaffold itself can also provide a sustained release system to effectively promote IPFSC migration (nearly twofold *in vitro*). Moreover, TGF-β3 loaded on scaffolds showed enhanced chondrogenic differentiation (such as collagen II, ACAN, and SOX9) of IPFSCs after 3 weeks of culture. After implanting the composite scaffold into the knee joints of rabbits, enhanced chondrogenic differentiation was discovered at 1, 2, and 4 weeks post-surgery, and improved repair of cartilage defects in terms of biochemical, biomechanical, radiological, and histological results was identified at 3 and 6 months post-implantation. To conclude, our study demonstrates that the growth factor (GF)-loaded scaffold can facilitate cell homing, migration, and chondrogenic differentiation and promote the reconstructive effects of *in vivo* cartilage formation, revealing that this staged regeneration strategy combined with endogenous cell recruitment and pro-chondrogenesis is promising for *in situ* articular cartilage regeneration.

## Introduction

Articular cartilage is a connective tissue that specifically adapts to harsh biomechanical environments; however, once injured, articular cartilage presents limited self-healing potential because it is devoid of blood supply, nerves and lymphatic tissues (Sophia Fox et al., 2009; Huey et al., 2012). Articular cartilage lesions caused by trauma, severe inflammation, infection and degenerative joint diseases predispose patients to joint pain or severe osteoarthritis (Wang et al., 2020; Yan et al., 2020). Conventional surgical treatments, such as microfracture (Steadman et al., 2003), autograft (Hangody and Füles, 2003), allograft mosaicplasty (Simon and Jackson, 2018), autologous chondrocyte implantation (ACI) (Richter et al., 2016), and even arthroplasty (Qiao et al., 2020), have been commonly proposed to repair such defects but cannot generate focal hyaline cartilage (Nie et al., 2020). Moreover, undesirable complications and a second operation are not uncommon (Chen et al., 2019a; Qiao et al., 2020). Tissue engineering for cartilage research provides biomaterial-based strategies to develop therapeutics for cartilaginous tissue growth and joint function restoration.

The approach of leveraging the body’s innate regenerative potential with biomaterials and bioactive cues to direct endogenous stem/progenitor cells to injured sites to assist with tissue repair is a recent trend in regenerative medicine (Gaharwar et al., 2020). Infrapatellar fat pad adipose-derived stem cells (IPFSCs), which reside in the site near articular cartilage, have attracted increasing attention due to their easy availability, rich quantity in autologous tissue, superior chondrogenic effects, less hypertrophy risk, inflammatory modulation, anti-senescence effects, cytokine secretion, and better scaffold culturing performance (Hindle et al., 2017; Zhong et al., 2020). However, the migration of IPFSCs as well as other endogenous MSCs naturally occurs during a short time window only and does not sufficiently repair the cartilage (Barry and Murphy, 2013; Yang et al., 2020). Therefore, an ideal scaffold for cartilage regeneration should provide a structural framework to facilitate endogenous MSC migration and drive the differentiation of these cells into cartilage-specific cell types.

Recently, Hakamivala et al. (2020) reported that erythropoietin (EPO)-loaded particles could effectively support cartilage regeneration by recruiting endogenous progenitor cells. However, the chondrogenic microenvironment for migrated cells is indeed. Therefore, multipotential growth factors (GFs) may be a better choice. TGF-β is a family of pleiotropic cytokines that regulate cell migration, proliferation, and differentiation, tissue repair and inflammation and are essential for cartilage formation (Makhijani et al., 2005; Qu et al., 2019). TGF-β3 has been reported to enhance stem cell migration, and a proof of concept study also showed that TGF-β3 facilitates articular cartilage formation *in vivo* on 3D-printed polycaprolactone (PCL) scaffolds (Gao et al., 2010; Lee et al., 2010). In addition, as a critical regulator of chondrogenic differentiation, TGF-β3 is also a potent GF that supports the chondrogenesis of MSCs *in vivo* and *in vitro* (Yang et al., 2017; Deng et al., 2019). TGF-β3 can also effectively induce collagen and proteoglycan synthesis by regulating the metabolism of articular cartilage and multipotent proteins in a time- and dose-dependent manner (Chen et al., 2019b). However, effective incorporation and controlled delivery of GFs remains a universal challenge for the clinical application of *in situ* tissue engineering strategies. Existing approaches rely on systemic or bolus injection and often cause administered GFs to rapidly diffuse away from the target site, leading to unwanted side effects. Given the pleiotropic effects of TGF-β3, it is vital to develop a delivery system to ensure controlled and localized release to the target tissues.

Acellular cartilage extracellular matrix (ECM) prepared by decellularization technology, which preserves active biological factors and maintains low immunogenic cellular components, has been reported to enhance cartilage regeneration and joint function recovery (Sutherland et al., 2015; Feng et al., 2020). As a biodegradable biomaterial, the ECM has been utilized to carry MSCs or chondrocytes for cartilage repair (Min et al., 2016; Li et al., 2019). Bioactive factors, such as chemokines or GFs, can also be incorporated into the ECM, which allows for continuous and local delivery of protein with the degradation of the ECM (Yang et al., 2017). Therefore, the ECM could be an excellent vehicle for GF delivery in cartilage tissue engineering. However, considering the inadequacy of the biomechanical properties of cartilage ECM-derived scaffolds, natural composite scaffolds developed for cartilage regeneration have the potential to overcome this problem (Yang et al., 2017). Demineralized cancellous bone (DCB), a natural 3D porous collagen network with excellent biocompatibility and mechanical strength, has been used as a scaffold for tissue-engineered musculoskeletal regeneration (Zhang et al., 2015; Yuan et al., 2016). Therefore, an ECM and DCB hybrid composite scaffold might be a potential construct to meet the treatment needs of cartilage defects.

**FIGURE 1:**
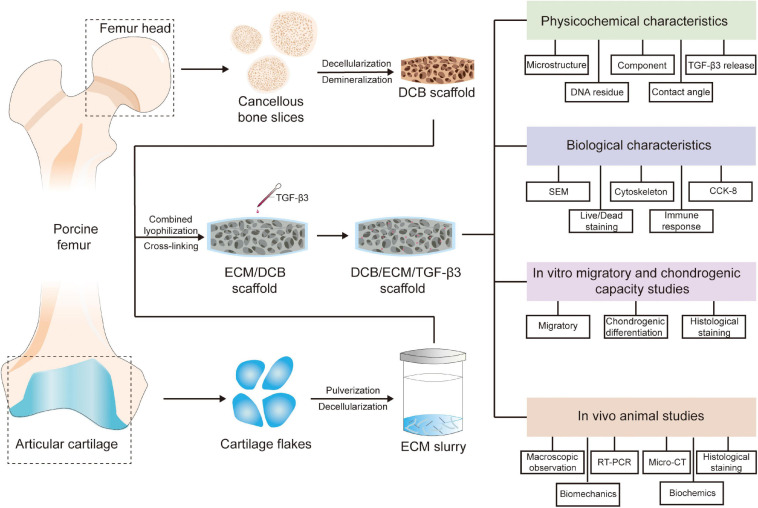
Schematic illustration of the overall study design. DCB, demineralized cancellous bone; ECM, extracellular matrix; TGF-β3, transforming growth factor-β3.

In this study, we used a lyophilization method to fabricate a TGF-β3-loaded DCB/ECM composite scaffold drug delivery system, which can integrate scaffolds and GFs for *in situ* cartilage tissue engineering (Figure 1). TGF-β3 exerts recruitment and chondrogenic effects simultaneously when released from scaffolds. We then tested the *in vitro* physicochemical properties and biocompatibility of the DCB/ECM scaffold, and *in vitro* recruitment and chondrogenic differentiation assays were performed. Finally, we implanted the composite scaffolds in a rabbit cartilage defect model to evaluate their therapeutic ability to promote *in situ* cartilage regeneration (Figure 1).

## Materials and Methods

### Preparation of Scaffolds

Extracellular matrix -coated porous DCB scaffolds were produced as previously reported (Yuan et al., 2016). DCB scaffolds were trimmed into a cylindrical shape (diameter: 3.5 mm; thickness: 1.2 mm) and were completely immersed in the ECM suspension accordingly (Table 1). After freezing and lyophilization, the DCB/ECM scaffolds were crosslinked using carbodiimide solution (14 mM 1-ethyl-3-(3-dimethylaminopropyl) carbodiimide hydrochloride [EDAC] and 5.5 mM *N*-hydroxysuccinimide [NHS]; Sigma) for 2 h and sterilized using ethylene oxide. Each DCB/ECM scaffold was perfused with 20 μL of 20 μg/mL TGF-β3 and incubated subsequently at 4°C for 20 min to form the DCB/ECM/TGF-β3 scaffold according to a previous study (Huang et al., 2018) (Figure 1).

**TABLE 1:** The compositions, pore size, and porosity of the scaffolds in this study.

Sample	ECM (wt%)	TGF-β3 (μg/mL)	Pore size (μm)	Porosity (%)
DCB scaffold	–	–	375.4 ± 38.52	84.93 ± 2.59
DCB/ECM scaffold	3%	–	67.76 ± 8.95****	71.04 ± 1.62*
DCB/ECM/TGF-β3 scaffold	3%	2	–	–

n = 5, *p < 0.05, ****p < 0.0001.

### Physicochemical Characterization of Scaffolds

#### Scanning Electron Microscopy

The surfaces and interior microstructural morphologies of the DCB and DCB/ECM scaffolds were characterized by scanning electron microscopy (SEM) (S-4800 field emission scanning electron microscope; Hitachi, Tokyo, Japan) observation after putter coating with gold. Subsequently, the pore size and porosity of the different scaffolds were calculated by the software Nano Measure1.2 (China) and ImageJ (United States), respectively, according to the SEM images.

#### Protein Release Behaviors

The *in vitro* release of TGF-β3 was determined by adding 1.0 mL PBS buffer (pH 7.4) containing 0.5% (w/v) bovine serum albumin (BSA) to proper scaffolds in Eppendorf tubes. Tubes were incubated in a shaking water bath (37°C, 60 rpm). At determined time intervals (1, 2, 4, 7, 14, 21, 28, 35, and 42 days), the extract (1 mL) was collected for analysis and replaced by isometric fresh PBS buffer. The percentage of released TGF-β3 was measured by ELISA (R&D Systems, United States) according to the manufacturer’s instructions. The ratio of cumulative release (in percent) was calculated based on the total amount of TGF-β3 obtained from the extracts.

#### Mechanical Testing

For compressive strength detection, approximately 5 mm cubes of the DCB and DCB/ECM scaffolds were tested using a BOSE biomechanical testing machine (BOSE 5100, United States). All scaffolds were kept moist in PBS buffer (pH 7.4) throughout these tests. The compression moduli were defined according to the slope of the linear fit to the strain-stress curves.

### Cytocompatibility, Immunogenicity and Cell Recruitment Study

#### Cell Viability Analysis

The viability of IPFSCs in the scaffolds was evaluated using a live/dead assay and SEM. After sterilization and washing in sterile PBS buffer, the scaffolds were seeded with 5 × 10^5^ IPFSCs in 20 μL DMEM/F12 (10% FBS) media and allowed to adhere for 2 h, during which 50 μL media was changed every 30 min; then, more media was added and refreshed every 2 days over the next 7 days.

The microstructure of the cell-scaffold composite and the growth of IPFSCs cultured *in vitro* on the scaffolds were observed by SEM. Cell-scaffold composites were harvested for 7 days after seeding. Specimens were fixed in 2.5% (v/v) glutaraldehyde and buffered with PBS. After putter coating with gold, the samples were observed using S-4800 field emission SEM (Hitachi, Tokyo, Japan).

Fluorescence staining of cells was observed by using a live/dead assay kit (Invitrogen, United States) after IPFSCs were seeded and cultured. After 4 days, scaffolds were washed with sterile PBS buffer and incubated in PBS solution with 2 mM calcein-AM and 4 mM ethidium homodimer-1 for 20 min at room temperature. Scaffolds were washed again with sterile PBS buffer, and images were acquired using a Leica TCS-SP8 confocal microscope (Leica, Germany) and analyzed with ImageJ software (United States). Cell viability was calculated as follows: (live cells/total cells) × 100% (*n* = 3).

#### *In vivo* Immune Responses Evaluation

Scaffolds were subcutaneously embedded into the back skin of SD rats to evaluate their *in vivo* biocompatibility. At 1 week after implantation, rats were euthanized, and H&E staining was performed to evaluate histological changes.

#### *In vitro* IPFSC Recruitment

**FIGURE 2:**
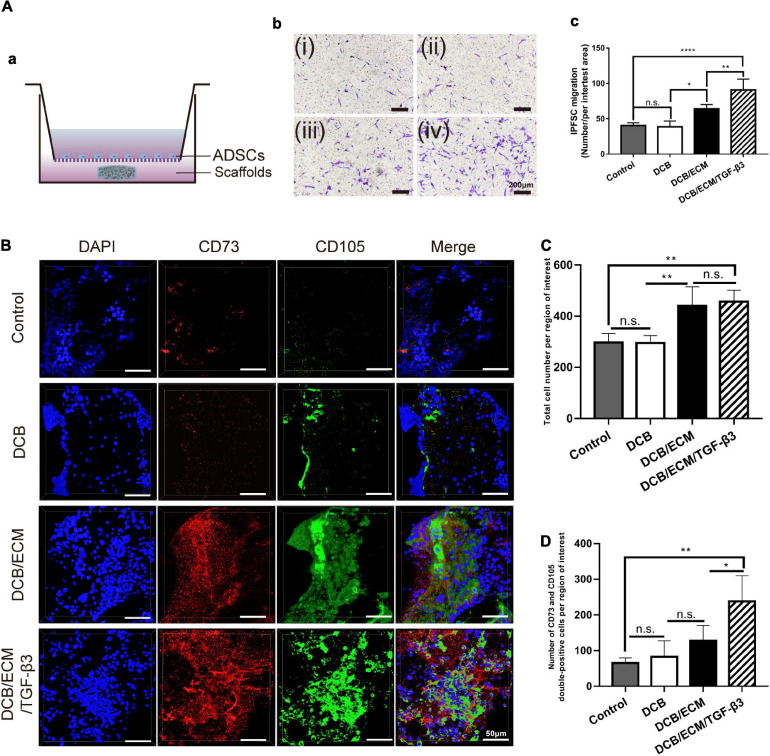
Migratory capacity of the different scaffolds on stem cells *in vitro* and *in vivo*. **(A)** Effects of different scaffolds on the migration of IPFSCs. **(a)** Schematic illustrations of the Transwell assay. **(b)** The migratory cells were stained with crystal violet after culturing for 24 h (i: negative control group; ii: DCB group; iii: DCB/ECM group; iv: DCB/ECM/TGF-β3 group). **(c)** Statistical analysis of the average migratory cell number per region of interest from different groups at the 24-h time point; values are presented as the means ± SDs (*n* = 5). **(B)** Confocal images of cell migration in the different scaffold groups (*n* = 5). **(C)** Total cell number migrated to the rat cartilage injured sites (*n* = 4). **(D)** Numbers of CD73/CD105 double-positive cells migrated to the rat cartilage injured sites (*n* = 4).

To determine the cell recruitment capability of the DCB/ECM/TGF-β3 scaffold on IPFSCs, a migration assay was performed according to the protocol described in the Supplementary Materials. DMEM (negative control), a DCB scaffold, a DCB/ECM scaffold and a DCB/ECM/TGF-β3 scaffold were added to the lower chamber (Figure 2A).

#### *In vivo* Endogenous MSC Recruitment Study in Rats

Sixteen SD rats were randomly allocated into four groups as follows: (A) negative control group, (B) DCB group, (C) DCB/ECM group, and (D) DCB/ECM/TGF-β3 group. A 2.0-mm diameter and 1-mm depth cartilage defect was created on the femoral trochlea of both limbs until there was slight bleeding. The different scaffolds were implanted at the defect site and the tissue and skin were sutured. At 7 days after the operation, rats were sacrificed, all debris was removed, and the distal femurs were collected. The MSC recruitment study was assessed by immunofluorescence staining (CD73, CD105, and DAPI).

#### *In vitro* Chondrogenic Differentiation

Chondrogenic differentiation was performed according to a previously published study (Fan et al., 2008). Approximately 4 × 10^5^ IPFSCs at passage 2 were centrifuged at 1500 rpm for 5 min in 15 mL Falcon tubes to form cell pellets. The pellets were maintained at 37°C with 5% CO_2_ in basal media for 24 h, after which they were placed in Transwell plates placed in 24-well plates. The 24-well plates contained either DCB scaffolds, DCB/ECM scaffolds, DCB/ECM/TGF-β3 scaffolds or nothing. Each well of the 24-well plates containing either the scaffold or nothing was nourished with chondrogenic induction media (CIM, Cyagen Biosciences, China). TGF-β3-free CIM consisted of basal medium supplemented with chondrogenesis supplementation (dexamethasone, ascorbate, insulin-transferrin-selenium solution, sodium pyruvate, proline). Medium was replenished every third day for 3 weeks. Chondrogenesis was qualitatively evaluated through H&E, toluidine blue, safranin O and collagen II immunofluorescence staining after 21 days (*n* = 4).

### *In vivo* Chondrogenic Differentiation Assay

#### Animal Surgery

**FIGURE 3:**
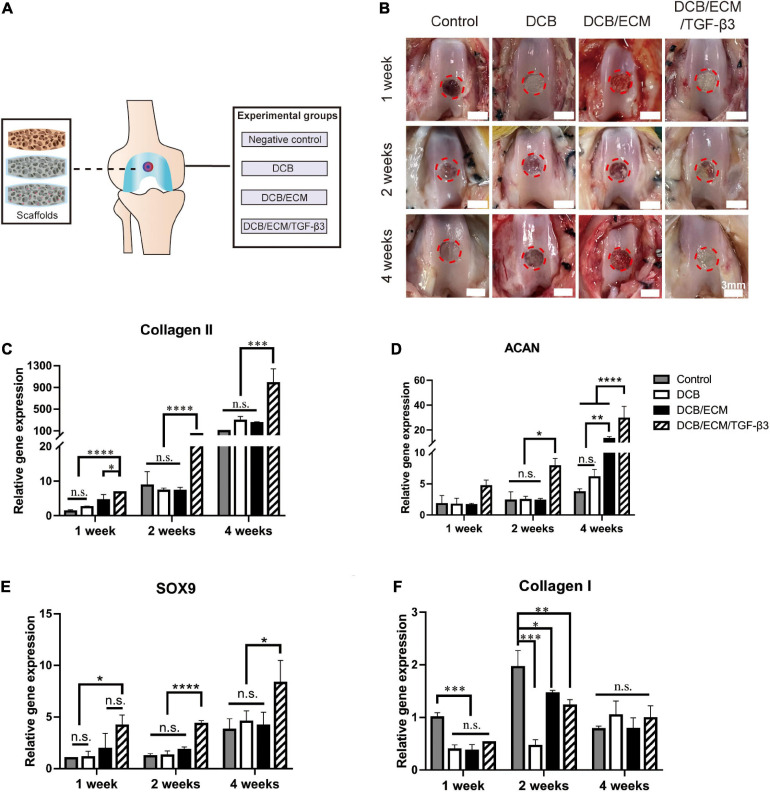
*In vivo* chondrogenic differentiation assay. **(A)** Scaffold implantation and experimental grouping schema. **(B)** Representative macroscopy of repaired tissues at 1, 2, and 4 weeks postsurgery. The red circles indicate the repaired areas. **(C–F)** Quantitative polymerase chain reaction (qPCR) analysis for collagen II, aggrecan (ACAN), transcription factor SOX9 and collagen I. The qPCR results were repeated three times independently. Data are means ± SDs (*p < 0.05, **p < 0.01, ***p < 0.005, ****p < 0.001, n.s. represents no significant difference).

An *in vivo* chondrogenic differentiation assay using a biofunctional scaffolding system was performed in a rabbit full-thickness cartilage defect model as described in our previous study (Li et al., 2019). All *in vivo* animal experiments were approved by the Institutional Animal Care and Use Committee at PLA General Hospital. This study used skeletally mature New Zealand White rabbits (male, weight 2.5–3.0 kg, 6 months old), and all animals were randomly allocated into four groups (*n* = 4 knees per group for each time point) as follows: (1) a negative control group, (2) a DCB group, (3) a DCB/ECM group, and (4) a DCB/ECM/TGF-β3 group (Figure 3A). In brief, we used a trephine to create a critical cartilage defect (3.5-mm in diameter and 1.2-mm in depth) on the patellar trochlear groove through the chondral layers. The defects of the experimental group were then implanted with three different scaffolds and adjusted to be flat against the surface of the surrounding cartilage. The negative control group received no scaffold treatment. After implantation, the joint capsule, subcutaneous tissue, and skin were closed, followed by intramuscular penicillin injections for up to 3 days. All rabbits were treated with the same dietary conditions, and none of them were excluded from this study. At different time points post-surgery, rabbits were euthanized and harvested for evaluation.

#### *In vivo* Chondrogenic Differentiation Assay

One week, 2 and 4 weeks post-implantation, a cylindrical tissue sample (3.5-mm in diameter and 1.2-mm in depth) was harvested from the defect site for further detection (*n* = 4 knees per group for each time point). Chondrogenic differentiation gene expression was analyzed using quantitative reverse transcription-polymerase chain reaction (RT-qPCR) as previously reported (Sun et al., 2018). Briefly, four independent cylindrical tissue specimens were snap-frozen in liquid nitrogen and then pulverized by a mortar. Total RNA was extracted using a standard TRIzol (Invitrogen, United States) procedure and quantified by a Nucleic Acid and Protein Analyzer (Microfuge18; Beckman-Coulter), followed by cDNA synthesis using a ReverTra Ace^®^ qPCR RT Kit (FSQ-201; TOYOBO). The specific gene primers designed for qPCR are listed in Table 2, and the experiment was performed using the StepOne TM Real-Time PCR System (Applied Biosystems). The relative gene expression was normalized to the housekeeping gene glyceraldehyde 3-phosphate dehydrogenase (GAPDH) and presented as the fold-change relative to the negative control group using the 2^ΔΔCt^ method.

**TABLE 2:** Primer sequences used for *in vivo* chondrogenic RT-qPCR.

Target gene		Sequence
SOX9	F: 5′-3′ R: 3′-5′	GCGGAGGAAGTCGGTGAAGAAT AAGATGGCGTTGGGCGAGAT
Collagen II	F: 5′-3′ R: 3′-5′	CACGCTCAAGTCCCTCAACA TCTATCCAGTAGTCACCGCTCT
Collagen I	F: 5′-3′ R: 3′-5′	GCCACCTGCCAGTCTTTACA CCATCATCACCATCTCTGCCT
ACAN	F: 5′-3′ R: 3′-5′	GGAGGAGCAGGAGTTTGTCAA TGTCCATCCGACCAGCGAAA
GAPDH	F: 5′-3′ R: 3′-5′	CAAGAAGGTGGTGAAGCAGG CACTGTTGAAGTCGCAG

### *In vivo* Cartilage Repair Study

#### Animal Surgery

*In vivo* cartilage repair using the biofunctional scaffolding system was assessed in the rabbit full-thickness cartilage defect model. All animal surgeries and groups were described above (Figure 3A). At 12 and 24 weeks post-surgery, all rabbits were euthanized and harvested for further detection (*n* = 8 knees per group for each time point).

#### Macroscopic Evaluation

All samples in each group of cartilage defects in the femoral condyles were observed by three independent evaluators and photographed (*n* = 8 knees per group for each time point). Macroscopic scoring was performed blindly by three experienced researchers specializing in musculoskeletal disease, following the ICRS scoring system guidelines.

#### Micro-CT Scanning

The samples were assessed using General Electric (GE) eXplorer Locus SP (GE, Boston, MA, United States) according to previous methods (Sun et al., 2018). The image data in the sagittal, frontal, and transverse planes were reconstructed and analyzed using GE Health Care MicroView ABA 2.1.2 software. A cylindrical region of interest (3.5-mm in diameter and 1.2-mm in depth) corresponding to the original defect location was selected to further assay. The BMD and BV/TV were then analyzed (*n* = 6 knees per group for each time point).

#### Biomechanical and Biochemical Assessment of Repaired Tissue

At 3 or 6 months postoperation, compressive strength detection was conducted according to the assessment of the biomechanical properties of repaired cartilage as described above (*n* = 3).

The neotissue total collagen content assay was performed by following the procedure described in the Supplementary Materials, and the collagen II content assay was performed by Western blot (WB). Every sample was cut into two equal parts for the above assays (*n* = 3 knees per group for each time point).

#### Histology and Immunohistochemistry

After examination by micro-CT, the samples were fixed in 4% PFA and then decalcified in 10% (w/v) EDTA (pH = 7.0) for 2 months at room temperature. Next, they were dehydrated and embedded in paraffin wax, sectioned into 6-μm slices and stained with H&E, toluidine blue, safranin O/fast green, and Sirius red according to the manufacturer’s protocols. Collagen II immunohistochemical staining was performed by immersing the sections into 0.25% pepsin (Abcam, United States) at 37°C for 20 min and blocking them in 10% goat serum for 1 h. After antigen retrieval, the slices were incubated with primary antibodies against collagen II (1:200; Developmental Studies Hybridoma Bank, United States) at 4°C overnight. After washing with PBS, they were incubated with goat anti-mouse IgG (1:200; Cat# NB7539; Novus) for 1 h. Finally, the sections were stained with Tris-HCl buffer containing 0.05% DAB and 0.005% hydrogen peroxide, and the nuclei were stained with hematoxylin. Photomicrographs were acquired using a Nikon microscope (Japan).

To evaluate the progress of subchondral bone reconstruction and cartilage repair, sections from three knees at 3 and 6 months per group (each sample represented three tissue sections) were blindly scored by three independent observers according to an established scoring system.

### Statistical Analysis

All quantitative data were analyzed using SPSS version 25.0 (SPSS, Chicago, IL, United States) and expressed as the mean ± standard deviation (SD). Student’s *t*-test, one-way analysis of variance (one-way ANOVA) or two-way ANOVA, followed by the Bonferroni multiple comparison test, was performed for normally distributed data. A value of *P* < 0.05 was considered to indicate a statistically significant difference.

## Results

### Physicochemical and Biological Characterization of Scaffolds

#### Scaffold Macro- and Microstructure

**FIGURE 4:**
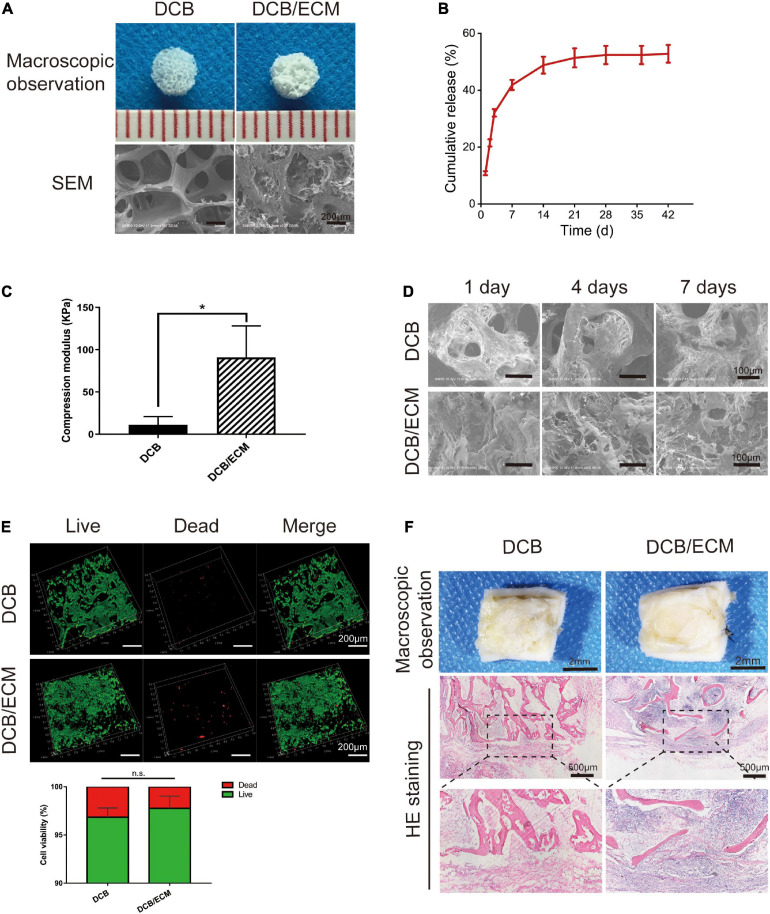
The physicochemical, biocompatibility, and immunogenicity properties of DCB and DCB/ECM scaffolds. **(A)** Macroscopic features and SEM of DCB and DCB/ECM scaffolds. **(B)** TGF-β3 release kinetics of the DCB/ECM/TGF-β3 scaffold. Values are presented as the means ± SDs (*n* = 3). **(C)** Compression modulus of DCB and DCB/ECM (scaffolds; values are presented as the means ± SD, *n* = 5). **(D)** SEM of DCB and DCB/ECM scaffolds on which IPFSCs were seeded for 1, 4, and 7 days. **(E)** Live/dead staining analysis of IPFSCs cultured in DCB and DCB/ECM scaffolds for 7 days. Representative 3D reconstruction images show live (green) cells and dead (red) cells. Values are presented as the means ± SDs (*n* = 3). (*p < 0.05, n.s. represents no significant difference). **(F)** Macroscopic observations and H&E staining of the immune responses of DCB and DCB/ECM scaffolds at 1-week postimplantation in rats.

Macroscopic observations of DCB and DCB/ECM scaffolds are shown in Figure 4A, and the results showed that DCB scaffold had a larger interconnected porous structure than the DCB/ECM scaffold. The SEM photographs of the DCB scaffold in Figure 4A showed circular pores in the size range of 375.4 ± 38.52 μm, while the DCB/ECM scaffold exhibited smaller irregular pores in the size range of 67.76 ± 8.95 μm (^****^p < 0.0001, *n* = 5, Table 1). The porosity of the two scaffolds was also calculated as follows: 84.93 ± 2.59% for DCB scaffold and 71.04 ± 1.62% for DCB/ECM scaffold (^∗^*p* < 0.05, *n* = 5, Table 1).

#### Protein Release Files

The TGF-β3-loaded DCB/ECM scaffolds were constructed according to the protocol (Figure 1). To assess the proteins released from the scaffold, total cumulative TGF-β3 release for 42 days was detected by enzyme-linked immunosorbent assay (ELISA) according to the manufacturer’s instructions. The DCB/ECM/TGF-β3 scaffolds released a cumulative rate of approximately 40% after 14 days and still increased up to 50% after 42 days (Figure 4B). These results suggest that the DCB/ECM scaffold could be a good drug release candidate with controlled and prolonged protein release kinetics for *in situ* tissue engineering.

#### Mechanical Characterization

To evaluate the biomechanical properties, a compressive strength assay was conducted to compare the DCB and DCB/ECM scaffolds. The compressive moduli of the DCB/ECM scaffold were superior (90.96 ± 37.22 kPa) to that of the DCB scaffold (11.34 ± 9.64 kPa, *n* = 5) (Figure 4C).

### Cytocompatibility and *in vivo* Immune Response of the Scaffolds

#### IPFSCs Attachment and Viability on the Scaffolds *in vitro*

The attachment of IPFSCs to DCB and DCB/ECM scaffolds was evaluated using SEM (Figure 4D). The IPFSCs attached to two scaffolds and migrated well into the interconnecting pores in DCB and DCB/ECM scaffolds over 1, 4, and 7 days culture periods. The IPFSCs were better distributed between interconnecting pores in the DCB/ECM scaffold during the three culture periods. This is probably because the pore sizes and hydrophilicity in the DCB/ECM scaffold were more suitable than those in the DCB scaffold, making it easier to attach to the interconnecting pores. To conclude, the inner walls of DCB and DCB/ECM scaffolds increase the surface area and might be suitable for IPFSC adhesion.

IPFSC viability on DCB and DCB/ECM scaffolds was observed by live/dead staining after 7 days of culture. For both scaffolds, most IPFSCs were stained with fluorescent green (living cells), with limited fluorescent red (dead) cells from 3D reconstruction images (Figure 4E). Quantitative cell viability analysis (*n* = 3) demonstrated that the cell viability rates on both the DCB and DCB/ECM scaffolds were higher than 96% but did not show any significant differences. The above results demonstrate that DCB and DCB/ECM scaffolds had good cytocompatibility and were suitable for cells to adhere and proliferate.

#### Scaffolds’ Immune Response in Rats

Acute inflammatory and immune responses of DCB and DCB/ECM scaffolds were evaluated at 1 week after rat subcutaneous implantation. No obvious scar tissues formation was found around the two scaffolds in the macroscopic observations and H&E staining images (Figure 4F). We observed some neutrophil and monocyte infiltration around the DCB/ECM scaffold, but only a small amount of immune cells were observed around the DCB scaffold, suggesting that the pure DCB scaffold had lower immunogenic properties than the DCB/ECM scaffold.

### *In vitro* Cell Recruitment and Chondrogenic Differentiation Assays

#### *In vitro* and *in vivo* Stem Cell Migration Assay

To determine the effect of different scaffolds on IPFSC mobility, we performed Transwell system assays *in vitro*. Twenty-four hours after stimulation with different scaffolds, the cell numbers were 40.25 ± 4.03 for the negative control group, 38.5 ± 8.35 for the DCB group, 64 ± 6.22 for the DCB/ECM group, and 90.75 ± 15.39 for the DCB/ECM/TGF-β3 group, among which the DCB/ECM/TGF-β3 group showed the best cell recruitment capacity (Figure 2A). To conclude, the above results indicate that the DCB/ECM and DCB/ECM/TGF-β3 scaffolds could promote IPFSC migration *in vitro* (*n* = 5, ^∗^*p* < 0.05, ^∗∗^*p* < 0.01, ^****^p < 0.001).

Moreover, *in vivo* MSC recruitment by TGF-β3 was further assessed by comparing the migrated MSCs in different groups at 1 week postoperation (Figure 2B). The results demonstrated that compared with the control and DCB groups, total cell numbers were higher in the DCB/ECM and DCB/ECM/TGF-β3 groups, while there were no dramatic differences between these two groups (Figure 2C). In addition, CD73 and CD105 double-positive cells were dramatically more concentrated in the DCB/ECM/TGF-β3 group than in the other groups (Figure 2D). These results also suggested that the TGF-β3 effectively enriched surrounding MSCs to the defect site and improved the regeneration of damaged cartilage.

#### *In vitro* Chondrogenic Differentiation Assay

**FIGURE 5:**
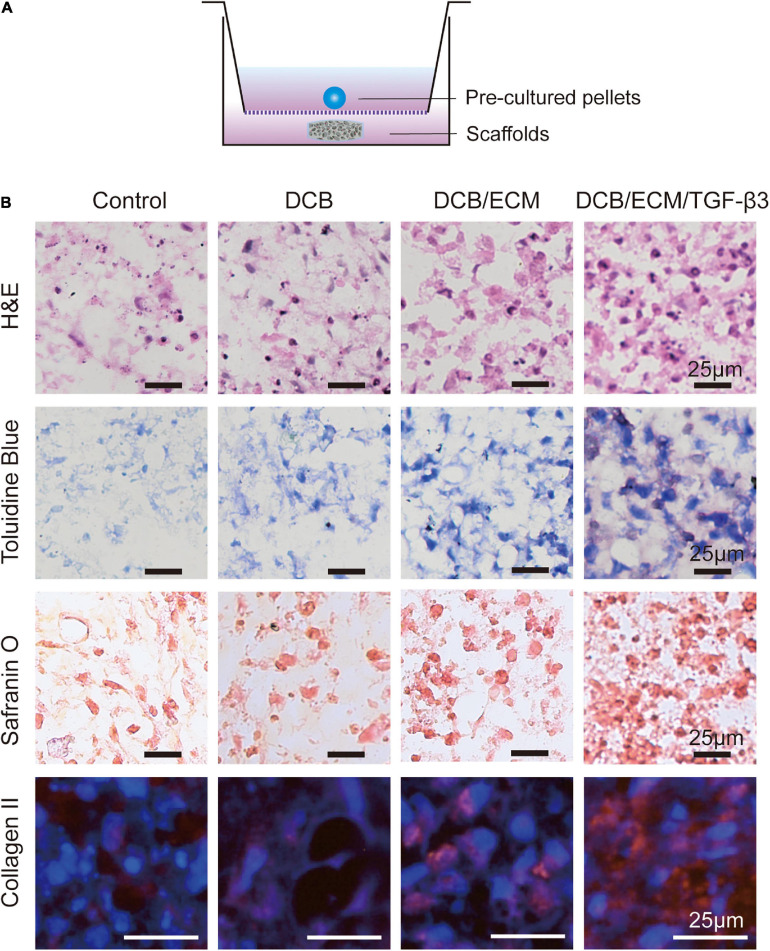
Chondrogenic capacity of the different scaffolds *in vitro*. **(A)** Schematic illustrations of the coculture systems between pre-cultured pellets and the different scaffolds. **(B)** Histological and immunofluorescence analyses of chondrogenic pellets performed in a coculture system with different scaffolds. H&E, toluidine blue, safranin O, and collagen II immunofluorescence staining were used.

To observe the bioactivity of TGF-β3 released from the scaffold, a 3D pellet coculture system experiment was performed according to previous studies (Figure 5A) (Chen et al., 2020). As shown in Figure 5B, H&E staining indicated that the 3D pellets were successfully cultured. Toluidine blue and safranin O staining, which stains synthesized proteoglycans, demonstrated the greatest intensity of pellets in the DCB/ECM/TGF-β3 scaffold group. In addition, collagen II immunofluorescence staining also showed that TGF-β3 released from scaffolds significantly promoted the secretion of collagen II.

### *In vivo* Chondrogenic Differentiation

After 1 week of *in vivo* implantation, gross observation demonstrated that cartilage defects were unrepaired in the control group and that scaffolds were still not completely degraded in the other three groups (control, DCB scaffold, and DCB/ECM scaffold). At 2 weeks, neocartilaginous tissue barely formed around the edge of the defects in the control, DCB and DCB/ECM groups. Moreover, cartilage defects in the DCB/ECM/TGF-β3 group were filled by a certain amount of repaired tissue (Figure 3B). After 4 weeks of implantation, cartilage defects were partially filled in the control group, but the cartilage did not regrow well. In the DCB and DCB/ECM groups, the defect was filled with regrown cartilage, but the surface was still rough. The regenerated cartilage in the DCB/ECM/TGF-β3 group was similar to the surrounding native cartilage tissue. However, obvious uneven edges between the surrounding cartilage still existed.

To demonstrate chondrogenic differentiation capabilities in different scaffolds, chondrogenic relative gene expression (collagen II, ACAN, SOX9 and collagen I) was assessed in the four groups at 1, 2, and 4 weeks postsurgery *in vivo* for the first time (Figures 3C–F). The results show that with time, the expression level of cartilage-related genes increased gradually, which indicates that chondrogenic differentiation occurs during the natural repair process and may play an important role in tissue regeneration. Furthermore, the expression levels of cartilage-related genes (collagen II, ACAN, and SOX9) were significantly upregulated in the TGF-β3-loaded DCB/ECM group; however, there were no significant differences among the control, DCB and DCB/ECM groups. This shows that supplementation with TGF-β3 could significantly stimulate chondrogenic differentiation of MSCs in defects compared with that in pure DCB scaffolds or DCB/ECM scaffolds. In terms of osteogenic differentiation, we did not find any regular trend of the related gene COL1 throughout the three different times *in vivo*. These results show that the scaffolds loaded with TGF-β3 could effectively enhance chondrogenic differentiation at the defect site and consequently enhance tissue repair and regeneration.

### *In vivo* Cartilage Repair Study

#### Gross Observation and Biomechanical Assessment of the Repaired Tissue

**FIGURE 6:**
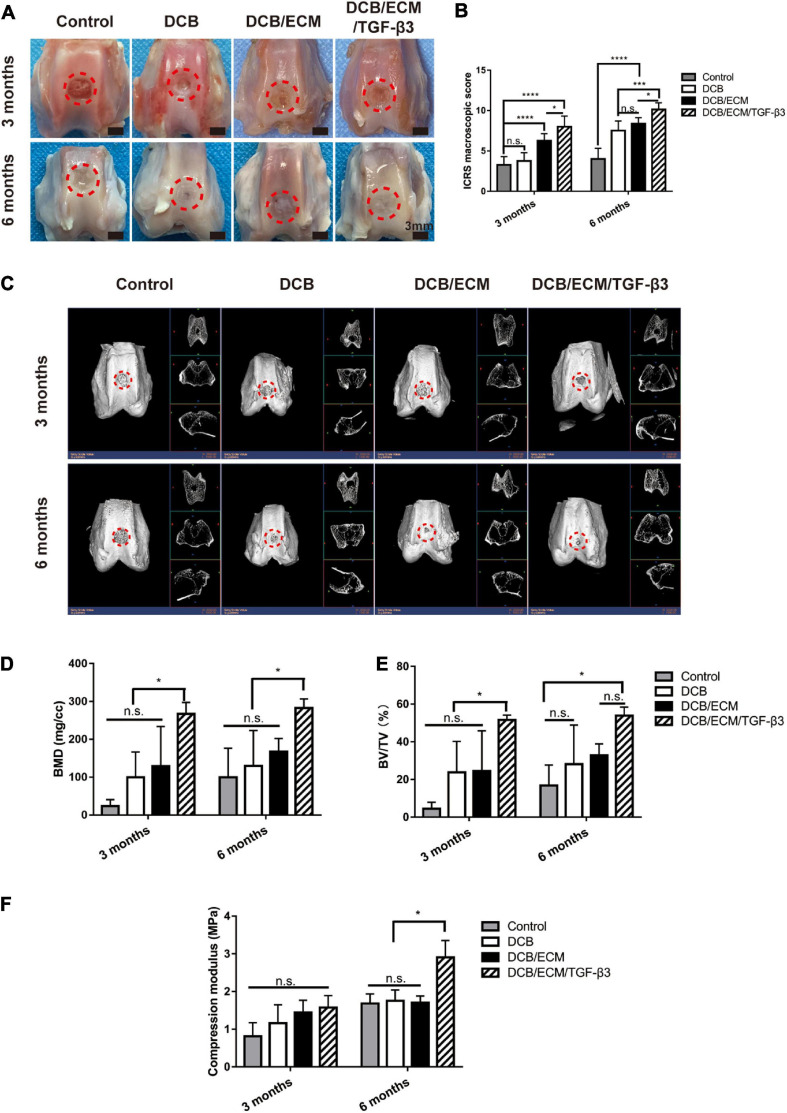
Representative macroscopic, radiological and biomechanical properties of repaired tissues at 3 and 6 months postoperation. **(A)** Representative macroscopy. **(B)** ICRS scores at 3 and 6 months. **(C)** Representative 3D and 2D micro-CT images at each time point. Quantitative analysis of **(D)** BMD and **(E)** BV/TV (*n* = 6 knees). The repaired sites were indicated by red circles. **(F)** Compression modulus of repaired tissues at 3 and 6 months (*n* = 3 knees). The red circles indicate the repaired areas. Data are means ± SDs (*p < 0.05, ***p < 0.005, ****p < 0.001, n.s. represents no significant difference).

The rabbit cartilage defect model was used to evaluate the therapeutic value of the scaffold. Three months postsurgery, gross observation demonstrated that cartilage defects were unrepaired in the control group (Figure 6A). In the DCB group, neocartilaginous tissue was partly formed surrounding the edge of the defects and showed irregular surface regularity with structural damage and fissures. Moreover, the cartilage defects in the DCB/ECM and DCB/ECM/TGF-β3 groups were filled with granulation tissue with uneven surfaces. We found that the regenerated cartilage of the DCB/ECM/TGF-β3 group was more similar to native cartilage than to that of the DCB/ECM group (Figure 6A). At 6 months, defects in the control group were characterized by incomplete filling of neotissue, surface irregularity, and distinct boundary areas. In the DCB group, the defect was filled with regrew cartilage, and cracks were observed in the center. In addition, cartilage defects were mostly filled in the DCB/ECM group. However, the surface was still rough, and the edges next to the surrounding cartilage were obviously uneven. The regenerated cartilage in the DCB/ECM/TGF-β3 group was similar to the surrounding native cartilage tissue, with a neat surface and complete fusion with the surrounding cartilage.

Consistent with the gross observation, the International Cartilage Research Society (ICRS) macroscopic scores of DCB/ECM/TGF-β3 (8.00 ± 1.31 at 3 months and 10.125 ± 0.84 at 6 months) were apparently better than those of the other groups: the control group (3.25 ± 1.04 at 3 months and 4.00 ± 1.31 at 6 months), DCB group (3.75 ± 1.04 at 3 months and 7.50 ± 1.20 at 6 months), and DCB/ECM group (6.25 ± 0.89 at 3 months and 8.38 ± 0.74 at 6 months) at both time points (^∗^*p* < 0.05, ^∗∗^*p* < 0.01, ^∗∗∗^*p* < 0.005, ^****^p < 0.001) (Figure 6B).

#### Microcomputed Tomography (Micro-CT) Analysis of the Repaired Tissue

For all groups, the growth pattern of the subchondral bone reconstruction at 3 and 6 months after surgery was evaluated by micro-CT imaging (Figure 6C). The quantitative bone mineral density (BMD) data were plotted (Figure 6D) and revealed that the value of the DCB/ECM/TGF-β3 group was significantly higher than that of the other three groups. Furthermore, no other significant differences within the control, DCB and DCB/ECM groups were found at any other time point. In addition, the bone volume-to-tissue volume ratio (BV/TV) values in the DCB/ECM/TGF-β3 group were dramatically higher than those in the other three groups at both time points (Figure 6E). In addition, only non-significant differences in BV/TV were found at 6-month time points between the DCB/ECM and DCB/ECM/TGF-β3 groups.

#### Biomechanical and Biochemical Assessment of Repaired Tissue

To evaluate the biomechanical properties of repaired tissue, compressive strength testing was conducted to compare the different groups. The 6-month repaired tissue generally had higher compressive moduli than the 3-month regenerated cartilage tissue. At 3 months postoperation, the compressive modulus was approximately 0.81 ± 0.36 MPa for the negative control group, 1.16 ± 0.49 MPa for the DCB group, 1.44 ± 0.32 MPa for the DCB/ECM group, and 1.51 ± 0.32 MPa for the DCB/ECM/TGF-β3 group (Figure 6F), whereas there was no significant difference among them. The compressive moduli of the repaired tissue (2.91 ± 0.45 MPa) in the DCB/ECM/TGF-β3 group were significantly higher than those in the other three groups (Figure 6F, *n* = 3, ^∗^*p* < 0.05).

**FIGURE 7:**
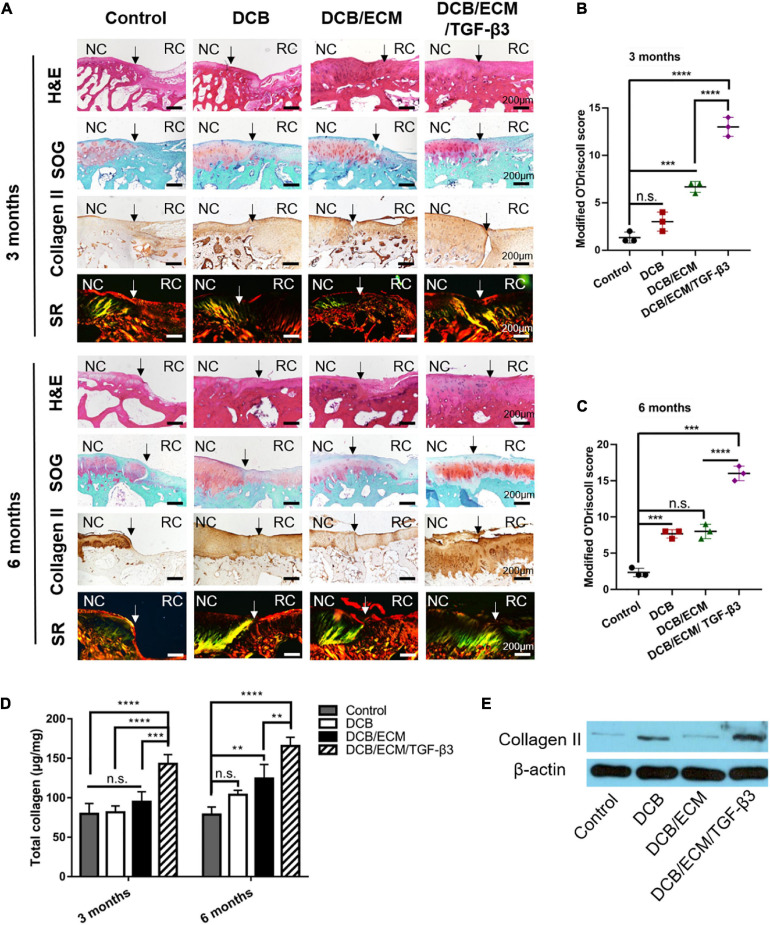
Histological and biochemical evaluation of repaired tissue. **(A)** Representative H&E, safranin O/fast green (SOG), collagen II and picrosirius red (SR) staining of repaired knees at 3 and 6 months (*n* = 6 knees). Staining was repeated twice or more independently. Modified O’Driscoll scoring for cartilage evaluation at 3 months **(B)** and 6 months **(C)** (*n* = 3 knees). **(D)** Total collagen contents of repaired knees at 3 and 6 months (*n* = 3 knees). **(E)** WB analysis of collagen II in repaired tissues at 6 months. NC, normal cartilage; RC, regenerated cartilage. Data are means ± SDs (**p < 0.01, ***p < 0.005, ****p < 0.001, n.s. represents no significant difference).

Biochemical assays for total collagen (Figure 7D) revealed that the total collagen content in the DCB/ECM/TGF-β3 group (142.90 ± 11.68 μg/mg at 3 months and 165.58 ± 10.92 μg/mg at 6 months) was significantly higher than that in the negative control group (79.61 ± 13.10 μg/mg at 3 months and 78.59 ± 9.66 μg/mg at 6 months), DCB group (81.59 ± 8.00 μg/mg at 3 months and 103.67 ± 5.80 μg/mg at 6 months), and DCB/ECM group (94.68 ± 12.87 μg/mg at 3 months and 124.21 ± 9.66 μg/mg at 6 months), among which the DCB/ECM group showed superior total collagen deposition than the negative control group (^∗∗^*p* < 0.01). In addition, the protein expression of collagen II (Figure 7E) in repaired tissue of the DCB/ECM/TGF-β3 group was also higher than that of the control group, DCB group and DCB/ECM group.

#### Histomorphometry of the Repaired Tissue

At 3 months, the histological staining results showed that the non-treated control group was insufficient to induce cartilage formation, the defect border remained, and fibrous tissues were filled (Figure 7A). In the DCB and DCB/ECM groups, distinct repaired tissue filled in the cartilage defect area, but these tissues were not properly integrated with adjacent cartilage. The deposition of proteoglycan and collagen II was also limited in this group. In contrast, the repaired tissue merged with the surrounding cartilage in DCB/ECM/TGF-β3 with abundant cartilaginous extracellular matrix deposition, which was strongly stained by an anti-collagen II antibody.

At 6 months, the defect area of the control group was not fully filled, and the repaired tissues were more likely fibrous tissue with poor proteoglycan content (Figure 7A). Compared to that at 3 months, the DCB and DCB/ECM groups produced more proteoglycan deposition and enhanced positive type II collagen on the surface of the defective joint. Indeed, the regenerated tissue in the DCB/ECM/TGF-β3 group presented with more type II collagen-enriched hyaline cartilaginous tissue retaining similarity to native cartilage. In addition, the deposition and organization of type II collagen in the defective site of each group were also assessed by Sirius red staining. The regenerated tissue in the DCB/ECM/TGF-β3 group showed higher collagen II expression, whereas other groups (control, DCB and DCB/ECM) tended to exhibit lower collagen II expression. Moreover, more organized collagen fibers were observed in the DCB/ECM/TGF-β3 group, presenting a more oriented pattern similar to hyaline cartilage.

After 3 and 6 months of treatment, a trend toward an elevated modified O’Driscoll score was noted in the DCB/ECM/TGF-β3 group (13 ± 1 at 3 months and 16 ± 1 at 6 months) compared to the other three treatment groups (control: 1.33 ± 0.58 at 3 months and 2.33 ± 0.58 at 6 months; DCB: 3 ± 1 at 3 months and 7.67 ± 0.58 at 6 months; DCB/ECM: 6.67 ± 0.58 at 3 months and 8 ± 1 at 6 months), particularly in the control group and DCB group (Figures 7B,C).

## Discussion and Conclusion

Recent advances in methods and materials have led to the development of suitable constructs for the clinical repair of injured cartilage (Cheng et al., 2019). However, the complex and multiple functions and limited self-healing capacity of native cartilage still hamper successful reconstruction of adult cartilage tissue restoration (Huey et al., 2012). Recently, *in situ* tissue engineering approaches that rely on recruiting endogenous cells to damaged sites avoid many drawbacks based on cell-seeded scaffolds and have offered great promise for *in situ* cartilage regeneration (Lee et al., 2010; Shi et al., 2017; Yang et al., 2020). To more effectively utilize the host’s own regenerative potential, a biofunctionalized scaffold with interconnecting and complex microchannels can serve as a platform for endogenous cell immobilization, infiltration and chondrogenesis (Lee et al., 2010). Lee et al. (2010) reported that TGF-β3-loaded 3D printed PCL/collagen composite scaffolds successfully regenerated the entire synovial articular cartilage surface of rabbits through host cell homing, diffusion, histogenesis, and angiogenesis. Another study conducted by Sun et al. (2018) proved that the cell-free scaffolding system DCM-RAD/SKP, which was produced by the integration of decellularized cartilage matrix (DCM) scaffold and self-assembly Ac-(RADA)4-CONH2/Ac-(RADA) 4GGSKPPGTSS-CONH2 (RAD/SKP) peptide nanofiber hydrogel, enhanced bone marrow-derived cell recruitment when combined with microfracture, thus facilitating articular cartilage regeneration.

Endogenous joint-resident cells play a critical role in joint pathophysiology, especially in cartilage injury (Yang et al., 2020). After cartilage damage, activated stem cells migrate and exert reparative effects via biochemical signals and finally differentiate into specialized cell types (Im, 2016; McGonagle et al., 2017). Notably, various subpopulations of endogenous stem/progenitor cells are critical for cartilage homeostasis and repair but may possess different potentials for chondrogenesis (Yang et al., 2020). Evidently, intraarticular fat pad-derived stem cells and synovium-derived MSCs were proven to be potent cell source reservoirs that contribute to chondrogenic differentiation and are less likely to lead to chondrocyte hypertrophy (Chen et al., 2015; Hindle et al., 2017). Therefore, it is necessary to introduce a bioactive factor to effectively recruit these cells to the injury site. However, rapid and uncontrolled release of GFs from biofunctionalized scaffolds at damage sites is probably unable to recruit a sufficient number of stem cells and has some unwanted side effects (Patel et al., 2019). This motivates tissue engineering researchers and clinicians to develop a multifunctional scaffold that can effectively carry and deliver GFs for sustained release. In this study, we aimed to test the reparative effects of a TGF-β3-loaded scaffold that combines articular cartilage ECM and DCB in cartilage regeneration. The results of the biochemical assays of residual DNA, total collagen and GAG (Supplementary Figures 2–4) and the histological and immunohistochemical staining (Supplementary Figure 5) indicated that the DCB/ECM scaffold showed a bionic structure and ingredients of native cartilage. In addition, *in vitro* cell migration experiments confirmed that TGF-β3- and TGF-β3-loaded scaffolds can facilitate IPFSC mobilization, which is in line with the results of previous studies (Lee et al., 2010) (Figure 3A and Supplementary Figure 9). The DCB/ECM scaffold showed a higher percentage water absorption (Supplementary Figure 1) and better surface hydrophilicity than the DCB scaffold (Supplementary Figure 6), and provided an optimal porous microenvironment for cell proliferation, infiltration and ECM production (Figures 4D,E and Supplementary Figures 7, 8). Moreover, *in vitro* and *in vivo* and chondrogenic experiments showed that the TGF-β3-loaded DCB/ECM scaffold exhibited superior chondrogenic capacity than the other scaffold without TGF-β3 (DCB scaffold and DCB/ECM scaffold) (Figures 3C–F, 5B).

An important point regarding the *in situ* tissue engineering strategies for cartilage regeneration involves a functional scaffold that can serve as a temporary “home” to (i) provide appropriate 3D structural and biomechanical support, (ii) facilitate resident stem cell migration, infiltration and proliferation, and (iii) initiate chondrogenic differentiation and stimulate ideal matrix deposition for functional cartilage regeneration. The natural microenvironment of the cartilaginous ECM plays an essential role in instructing cell fate, mainly owing to its microstructures and bioactive contents (Sun et al., 2018; Kim et al., 2019). Hence, cartilage ECM-based materials can act as a more suitable microenvironment to better mimic natural cell-ECM interactions and further improve cartilage repair outcomes. Previous studies have shown that DCBs contain a natural 3D porous structure and exert excellent biocompatibility and promising mechanical properties, thus ideally combining with the cartilage ECM to spontaneously mimic the 3D microenvironment and provide biomechanical support (Yuan et al., 2016; Kim et al., 2019). In our study, both SEM, live/dead and DAPI/phalloidin staining confirmed that DCBs and DCB/ECM possess proper microstructure and biocompatibility (Figures 4D,E and Supplementary Figure 8). The introduced GF TGF-β3 has been shown to be capable of recruiting approximately 130% more endogenous stem cells to cartilage regenerated sites (Lee et al., 2010). However, bolus injection of GFs tends to induce rapid diffusion and inflammatory side effects. When absorbed and released from the DCB/ECM delivery platform, *in vitro* release experiments demonstrated prolonged release profiles (Figure 4B) and could also significantly modulate and facilitate IPFSC mobilization (Figure 2A and Supplementary Figure 9). Considering the above, the DCM/ECM scaffold acts as a biofunctional “home” for stem cell resistance and delivery of bioactive factors that improve cell recruitment and chondrogenesis.

To validate the biodegradability and chondrogenic effects of the biofunction-composited scaffold, *in vivo* degradability and chondrogenic experiments were performed (Figure 3). After 1, 2, and 4 weeks of *in vivo* implantation, we harvested and captured the *in situ* degradation and repair performance of each group and demonstrated that DCB/ECM/TGF-β3 had excellent biodegradability to orchestrate neotissue ingrowth (Figure 3B). Next, we demonstrated that the TGF-β3-loaded DCB/ECM scaffold exhibited more chondrogenic-related gene expression than the scaffold without additional GFs. Significantly, in the DCB/ECM/TGF-β3 group, more collagen II, ACAN and SOX9 expression at all time points was demonstrated (Figures 3C–F). Additionally, collagen I expression in the control group was highest at 1 and 2 weeks after implantation, which means that the repaired tissue was more fibrous-like. These results suggest that the GF-functionalized DCM/ECM scaffold possesses favorable biodegradability and provides a suitable and inductive host microenvironment for chondrogenesis of migrated cells. On the basis of our cartilage layer defect animal model, the recruited MSCs might be mainly derived from intraarticular fat pats, synovial tissue, synovial fluid, or even the vascular system. However, the lack of evidence from *in vivo* recruitment experiments and limited knowledge of cell markers hampered our understanding of certain participants; thus, further studies need to be conducted to understand the subpopulations of these cells in cartilage regeneration.

In terms of *in vivo* cartilage repair studies, DCB, DCM/ECM, and DCB/ECM/TGF-β3 scaffolds can promote cartilage repair to different extents. Histomorphometry (Figure 7), radiographic (Figures 6C–E), and biomechanical assessment (Figure 6F) analyses confirmed that the DCB/ECM/TGF-β3 scaffold showed superior repair results in terms of histological structure, biochemical contents, biomechanical performance and subchondral bone reconstruction. Although neocartilage could be observed in the control groups, it was quite inferior to that in the TGF-β3-loaded group. This may be because the natural composite scaffold could only provide structural support but was not inductive enough for cell infiltration and chondrogenesis.

**FIGURE 8:**
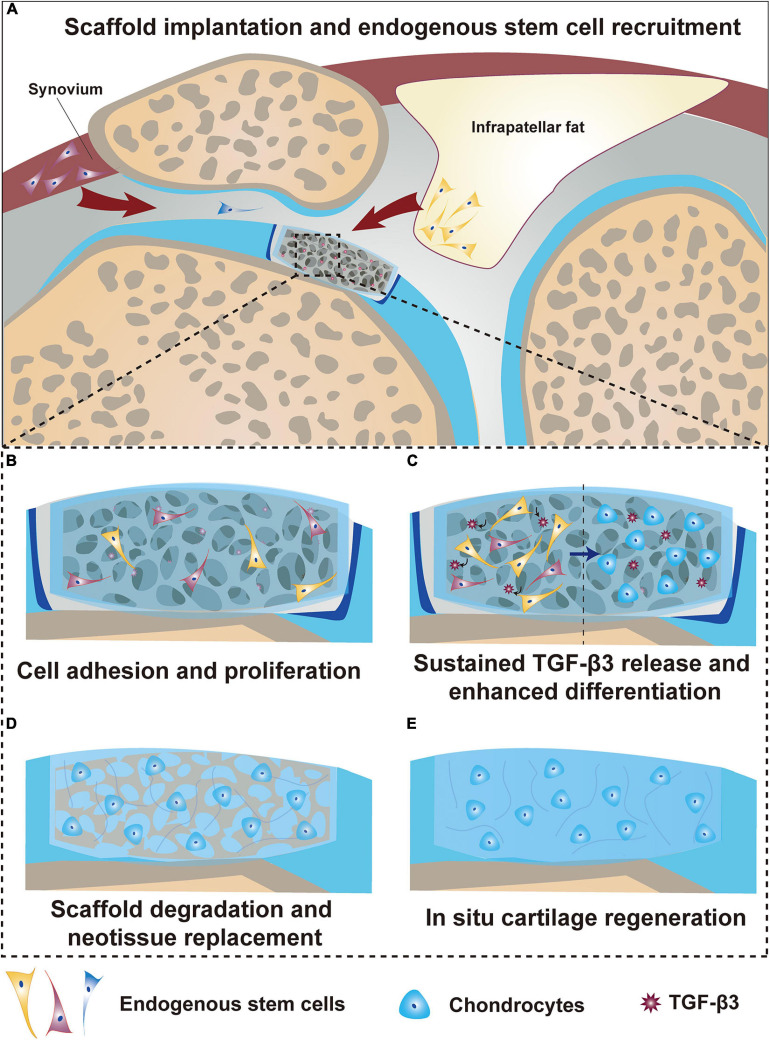
Summarized schematic of the mechanism of *in situ* cartilage regeneration. **(A)** Scaffold implantation and endogenous stem cell recruitment. **(B)** Stem cell adhesion and proliferation. **(C)** Sustained TGF-β3 release and enhanced stem cell differentiation. **(D)** Scaffold degradation and neotissue replacement. **(E)** *In situ* articular cartilage regeneration.

We proposed a possible mechanism of cartilage regeneration based on the findings of the present study (Figure 8). First, when a biofunctionalized scaffold was implanted into the cartilage defect, the fast released TGF-β3 acted as a signaling molecule to recruit resident stem cells within the joint to infiltrate into the scaffold (Figure 8A). Then, various adhesion proteins, GFs and the hydrophilic surface of this composite decellularized constructure enabled cells to adhere and proliferate well around every corner within the scaffold and interface (Figure 8B). Additionally, the prolonged release of TGF-β3 cooperated with biomechanical stimuli to induce chondrogenic differentiation of recruited and proliferated cells in the targeted space (Figure 8C). Finally, scaffold degradation was orchestrated with neotissue replacement and achieved optimal remodeling, mutation and regeneration of the cartilage (Figures 8D,E).

We must admit that there are some limitations to this study. First, the *in vitro* release of TGF-β3 lasted for only 6 weeks, which falls short of the *in vivo* repair requirement. On the other hand, investigations of this DCB/ECM/TGF-β3 scaffold in larger animals at a longer time point may be more clinically relevant. Although this study presents promising results in cartilage regeneration, there still remains a significant challenge for clinical translation. Briefly, our study provides a potential scaffolding system with many advantages for one-step surgical implantation such as availability, low immunogenicity and biodegradability. Therefore, our scaffolds and related regeneration strategies may not only provide new curative options for articular cartilage regeneration but also avoid laborious effort in contrast to *in vitro* cell culture prior to *in vivo* implantation.

In conclusion, the present study developed a staged regeneration strategy that combines endogenous cell recruitment and pro-chondrogenesis approaches for *in situ* articular cartilage regeneration. As a proof of concept, we created a 3D hybrid DCB/ECM/TGF-β3 scaffold with biomimetic microarchitecture and bioactivity through a combination of dual-functional TGF-β3 enhancing reparative cell recruitment and chondrogenic differentiation and a DCB/ECM scaffold with biomimetic microarchitecture facilitating reparative cell settlement and proliferation. The biofunctionalized scaffold has been proven to recruit IPFSCs *in vitro* and support the cell settlement and chondrogenic differentiation of migratory cells. Our *in vivo* analysis also demonstrated that the functional scaffold could promote superior cartilage regeneration and subchondral bone protection in a rabbit full-thickness cartilage defect model. In conclusion, with the help of controlled and prolonged drug delivery, this staged regeneration strategy, which leverages the body’s innate regenerative potential, holds great promise for clinically effective *in situ* articular cartilage regeneration.

## Data Availability Statement

The raw data supporting the conclusions of this article will be made available by the authors, without undue reservation.

## Ethics Statement

The animal study was reviewed and approved by Institutional Animal Care and Use Committee at PLA General Hospital.

## Author Contributions

ZY: writing original draft, investigation, data curation, formal analysis, and visualization. HL: investigation and data curation. YT: investigation and visualization. LF: data curation and formal analysis. CG: data curation. TZ: formal analysis. FC: methodology. ZL: software. ZY: conceptualization and supervision. SL: supervision and project administration. QG: supervision. All authors contributed to the article and approved the submitted version.

## Conflict of Interest

The authors declare that the research was conducted in the absence of any commercial or financial relationships that could be construed as a potential conflict of interest.
