# Supplementary material for: Biofunctionalized Structure and Ingredient Mimicking Scaffolds Achieving Recruitment and Chondrogenesis for Staged Cartilage Regeneration
Source: Front Cell Dev Biol. 2021 Mar 25;9:655440. doi: 10.3389/fcell.2021.655440 (PMC8027342; doi:10.3389/fcell.2021.655440)
Supplement: Supplementary file 1 [file Data_Sheet_1.docx]

**Supplementary data**

**Biofunctionalized structure and ingredient mimicking scaffolds achieving recruitment and chondrogenesis for staged cartilage regeneration.**

Zhen Yang^1,2†^, Hao Li^1,2†^, Yue Tian^1†^, Liwei Fu^1,2^, Cangjian Gao^1,2^, Tianyuan Zhao^1,2^, Fuyang Cao^1,3^, Zhiyao Liao^1,2^, Zhiguo Yuan^4^*, Shuyun Liu^1^*, Quanyi Guo^1,2^*

1: Institute of Orthopedics, the First Medical Center, Chinese PLA General Hospital; Beijing Key Lab of Regenerative Medicine in Orthopedics; Key Laboratory of Musculoskeletal Trauma & War Injuries PLA; No. 28 Fuxing Road, Haidian District, Beijing 100853, China

2: School of Medicine, Nankai University, Tianjin 300071, China

3: Department of Orthopedics, the First Affiliated Hospital of Zhengzhou University, 1 Jian East Road, Eqi District, Zhengzhou 450052, China

4: Department of Bone and Joint Surgery, Renji Hospital, School of Medicine, Shanghai Jiaotong University, Shanghai, China

***: Corresponding authors:** (1) Guo Quanyi, Institute of Orthopedics, The First Medical Center, Chinese PLA General Hospital, Beijing Key Lab of Regenerative Medicine in Orthopedics, Key Laboratory of Musculoskeletal Trauma and War Injuries PLA, No. 28 Fuxing Road, Haidian District, Beijing 100853, China. School of Medicine, Nankai University, Tianjin 300071, China. Email address: doctorguo_301@163.com.

(2) Liu Shuyun, Institute of Orthopedics, The First Medical Center, Chinese PLA General Hospital, Beijing Key Lab of Regenerative Medicine in Orthopedics, Key Laboratory of Musculoskeletal Trauma and War Injuries PLA, No. 28 Fuxing Road, Haidian District, Beijing 100853, China. Email address: clear_ann@163.com.

(3) Yuan Zhiguo, Department of Bone and Joint Surgery, Renji Hospital, School of Medicine, Shanghai Jiaotong University, Shanghai, China. Email address: yzgad@163.com.

†: These authors contributed equally.

**Materials and Methods**

**1. Preparation and assessment of DCB and ECM**

**1.1 Preparation of cartilage ECM**

Acellular cartilage extracellular matrix (ECM) slurry was physically-chemically extracted from swine as previously described with some modifications [1]. Briefly, the cartilage tissue was harvested from a swine knee joint. After washing in phosphate-buffered saline (PBS, Sigma, USA) and sterilizing with 3% H_2_O_2_, the minced cartilage was decellularized according to the differential centrifugation method: 2000 rpm for 30 min, 3000 rpm for 30 min, and 4000 rpm for 30 min. After the procedures were repeated 5 times, the ECM slurry was centrifuged at 10000 rpm for 30 min at 4 °C. After decellularization, the acellularized articular cartilage ECM slurry was stored at 4 °C.

**1.2 Preparation of demineralized cancellous bone (DCB)**

The production of the DCB scaffold was described previously [2, 3]. Briefly, we dissected swine femur epiphyses and immersed them in electrolyzed oxidizing water for 24 h, followed by 3% hydrogen peroxide (H_2_O_2_) for 24 h, 0.5% acetic acid treatment for 24 h and washing with distilled water. For defat treatment, these samples were immersed in a 1:1 (v/v) mixture solution of chloroform and methanol for 48 h. Then, the defatted samples were demineralized for 28 days at 25 °C in ethylene diamine tetra acetic acid (EDTA; 10% v/w, pH = 7.0) solution, which was changed every 3 days. The demineralized samples were washed totally with distill water and freeze-dried before the in vitro or in vivo experiments.

**1.3 Water absorption**

The water absorption capacity of the DCB and DCB/ECM scaffolds were measured by weighing the freeze-dried and swollen scaffolds. In brief, dry scaffolds were weighed [W_d_] then rehydrated in PBS buffer at 37 °C for 24 h to reach an equilibrium swelling state. Then, the wet scaffolds were removed from PBS and gently blotted with blotting paper to remove the residual liquid and weighed [W_s_]. Water uptake was calculated according to the following equation:

Qs=[(W_s_-W_d_)/ W_d_] × 100%

**1.4 Histological and immunohistochemical staining**

DCB and DCB/ECM scaffolds were fixed in 4% (v/v) para-formaldehyde solution (PFA) (Sigma, USA) and sectioned into 6-μm frozen slices. Scaffolds were stained with hematoxylin-eosin (H&E) and sulfated proteoglycans were stained with toluidine blue and safranin-O. For immunohistochemical staining with monoclonal antibodies against collagen I and Ⅱ (Novus, USA), the sections were treated with 3% (v/v) H_2_O_2_ for 30 min, incubated with primary antibodies overnight at 4 °C before being washed and incubated with horseradish-conjugated secondary anti-bodies (1:150; Cat# NB7539; Novus). Finally, the section’s signal was developed using diaminobenzidine (DAB) (Beijing Zhongshan Jinqiao Biological Technology Co., Ltd., China) and nuclei were counterstained with hematoxylin.

**1.5 Biochemical Assays**

The DNA in the DCB and DCB/ECM scaffolds was extracted and quantified using a TIANamp Genomic DNA kit (TIANamp, China) and PicoGreen DNA assay kit (Invitrogen, Carlsbad, CA, USA), respectively. The collagen and glycosaminoglycan (GAG) contents were quantified using a hydroxyproline assay kit (Nanjing Jiancheng, China) and DMMB Colorimetry kit (Genmed Scientific Inc., Shanghai, China), respectively. All the experiments were performed according to the manufacturer’s instructions.

**1.6 Hydrophilic characteristic**

A droplet of distilled water was dropped onto the surface of the DCB and DCB/ECM scaffolds (1 cm^3^) at a controlled rate and then the contact angles between water and scaffolds’ surface at different times were captured and measured using a tensiometer (Dataphysics OCA20, Germany) at room temperature.

**1.7 Scaffolds’ immune response**

Scaffolds were subcutaneously embedded into the back skin of SD rats to evaluate their in vivo biocompatibility. At 1 week after implantation, rats were euthanized, and H&E staining was performed to evaluate the histological changes.

**2. Cytocompatibility and cell recruitment in vitro study**

**2.1 Cell culture**

IPFSCs were isolated according to previous studies [4, 5]. In brief, the sub-patellar fat pad of knee joint adipose tissue was obtained and thoroughly washed three times with PBS containing 1% penicillin-streptomycin (Sigma, USA) and then cut into fine sections (no larger than 1 mm^3^) using scissors. Then, the cut adipose tissue was transferred into Dulbecco's modified Eagle’s medium/F12 (DMEM/F12, Corning, USA) containing 0.25% of collagen solution I, placed in the incubator at 37 °C for 2 h and centrifuged at 1500 g for 10 min. Finally, the cells were resuspended in DMEM/F12 containing 10% fetal bovine serum (FBS, Gibco, USA) and subsequently cultured in 25 cm^2^ flasks (Corning, USA). The cells (80-90%) were progressed to passage after cell fusion. The passage 2-5 of cells was chosen to conduct the following studies.

**2.2 Cell proliferation assay**

Cell counting kit-8 reagent (CCK-8; Dojindo, Japan) was used to quantitatively measure the proliferation of the stem cells seeded on scaffolds (2 × 10^3^ cells/scaffold). After incubation for 1, 4 or 7 d, the cell-seeded scaffolds were relocated to a new 96-well plate with the working solution (CCK-8 reagent/cell culture medium=volume ratio of 1:10). Scaffold-free plates were used as blanks. After incubating at 37 °C for 2 h, the absorbance of the test solutions (n=5/group) was detected at 450 nm using a microplate reader (Beckman, Fullerton, CA).

**2.3 Cytoskeleton staining**

In terms of the evaluation the cell cytoskeleton seeded on scaffolds, the scaffolds were stained by phalloidin (Cytoskeleton, USA) and 4',6-diamidino-2-phenylindole (DAPI) after 4 d of culture according to the manufacturer’s instructions. Scaffolds were washed again with sterile PBS buffer and images were acquired using a Leica TCS-SP8 confocal microscope (Leica, Germany) and analyzed with ImageJ software (USA).

**2.4 In vitro IPFSC recruitment**

To determine the concentration of TGF-β3 on the migration of IPFSCs, a migration assay was performed using the Transwell system (Corning, USA) as previously described. Briefly, 2×10^3^ IPFSCs were seed at the bottom of the upper chamber, and one of the following solutions was added to the lower chamber: 600 μL DMEM (negative control), 1 ng/mL TGF-β3+DMEM, 10 ng/mL TGF-β3+DMEM, or 100 ng/mL TGF-β3+DMEM (Figure S9). Twenty-four h after seeding, IPFSCs were fixed with 4% PFA for 20 min and stained with 0.2% methylrosanilnium chloride solution (Sigma, USA) for 10 min at room temperature. IPFSCs in five randomly selected fields at 200× magnification were analyzed.

**Results**

**1.1 Water absorption**

The DCB/ECM scaffold (1407.68 ± 83.05%) showed a higher percentage water absorption than the DCB scaffold (822.66 ± 39.70%, n=5) (Figure S1).

**1.2 Biochemical Assays**

To explore whether residual nuclei remained in both the DCB and DCB/ECM scaffolds, we extracted and quantified the DNA in each scaffold according to the manufacturer’s instructions. The results, as shown in Figure S2, demonstrated that the concentration of residual DNA was less than 20 ng/mg (n=3), demonstrating that most of the host cells were removed after the decellularization process.

The biochemical analysis of total collagen and GAG in the DCB and DCB/ECM scaffolds is shown in Figure S3 and Figure S4. There was no significant difference in total collagen content between the two groups (DCB: 215.54 ± 9.74 μg/mg and DCB/ECM: 189.11 ± 19.96 μg/mg), but the DCB/ECM scaffold showed a lower collagen content than the native cartilage (246.21 ± 27.80 μg/mg, *p<0.05, n=3). In addition, the GAG content in the DCB/ECM scaffold (4.86 ± 0.18 μg/mg) was higher than that in the DCB scaffold (0.86 ± 0.77 μg/mg), while it showed no significant difference compared with the native cartilage (n=3, **p<0.01).

**1.3 Histological and immunohistochemical staining**

Both the DCB and DCB/ECM scaffolds were assessed by histological and biochemical analyses (Figure S5). H&E staining showed that both the DCB and DCB/ECM scaffolds possessed good circular morphology. Toluidine blue and safranin O staining showed higher positive results in the DCB/ECM scaffold than in the DCB scaffold, indicating that the GAG content derived from the articular cartilage ECM remains after decellularization. Immunochemical staining (Figure S5) showed abundant collagen II in the DCB/ECM scaffold, while only a small amount of collagen II was found in the DCB scaffold. In addition, these results also demonstrated that residual collagen I was restricted to the DCB scaffold.

**1.4 Contact angle assessment**

The contact angles decreased after distilled water as dropped onto the surface of the DCB and DCB/ECM scaffolds. There were no significant differences in the contact angles between the two groups at 5 s in Figure S6 (DCB: 102.40° ± 2.85°; DCB/ECM: 100.95° ± 3.93°, n=4). However, the contact angle for the DCB/ECM scaffold was reduced after 5 s of contact time and showed a significant difference with the DCB scaffold (Figure S6), indicating that the DCB/ECM scaffold had better surface hydrophilicity.

**1.5 In vitro cell proliferation**

CCK-8 quantitative assay (Figure S7) was used to explore the capability of the DCB and DCB/ECM scaffolds to promote cell proliferation. The results demonstrated that the absorbance values of both scaffolds increased with the incubation time. Along with the control group, both scaffolds could also promote IPFSC proliferation without any cytotoxicity features.

**1.6 In vitro biocompatibility evaluation**

In addition, 3D reconstruction phalloidin staining images displayed that IPFSCs adhered and grew well in both the DCB and DCB/ECM scaffolds (Figure S8). The above results demonstrate that the DCB and DCB/ECM scaffolds had good cytocompatibility and were suitable for cells to adhere and proliferate.

**1.7 In vitro cell migration assay**

To determine the effect of different concentrations of TGF-β3 on IPFSC mobility, we performed a Transwell system assay in vitro. Twenty-four h after different concentrations of TGF-β3 stimulation, the migrated cell numbers of the 1 ng/mL TGF-β3+DMEM, 10 ng/mL TGF-β3+DMEM, and 100 ng/mL TGF-β3+DMEM groups were 66.00 ± 11.02, 69.20 ± 7.43, and 79.60 ± 4.45, respectively, which showed significant differences compared to the control group (48.20 ± 4.21) (Figure S9).

**References**

[1] Q. Yang, J. Peng, Q. Guo, J. Huang, L. Zhang, J. Yao, F. Yang, S. Wang, W. Xu, A. Wang, A cartilage ECM-derived 3-D porous acellular matrix scaffold for in vivo cartilage tissue engineering with PKH26-labeled chondrogenic bone marrow-derived mesenchymal stem cells, Biomaterials 29(15) (2008) 2378-2387.

[2] Z. Yuan, S. Liu, C. Hao, W. Guo, S. Gao, M. Wang, M. Chen, Z. Sun, Y. Xu, Y. Wang, AMECM/DCB scaffold prompts successful total meniscus reconstruction in a rabbit total meniscectomy model, Biomaterials 111 (2016) 13-26.

[3] H. Huang, X. Hu, X. Zhang, X. Duan, J. Zhang, X. Fu, L. Dai, L. Yuan, C. Zhou, Y. Ao, Codelivery of Synovium-Derived Mesenchymal Stem Cells and TGF-β by a Hybrid Scaffold for Cartilage Regeneration, ACS Biomaterials Science & Engineering 5(2) (2018) 805-816.

[4] H. Cheng, Y. Zhang, B. Zhang, J. Cheng, W. Wang, X. Tang, P. Teng, Y. Li, Biocompatibility of polypropylene mesh scaffold with adipose-derived stem cells, Experimental and Therapeutic Medicine 13(6) (2017) 2922-2926.

[5] S.-C. Wu, C.-H. Chen, J.-Y. Wang, Y.-S. Lin, J.-K. Chang, M.-L. Ho, Hyaluronan size alters chondrogenesis of adipose-derived stem cells via the CD44/ERK/SOX-9 pathway, Acta Biomaterialia 66 (2018) 224-237.

**
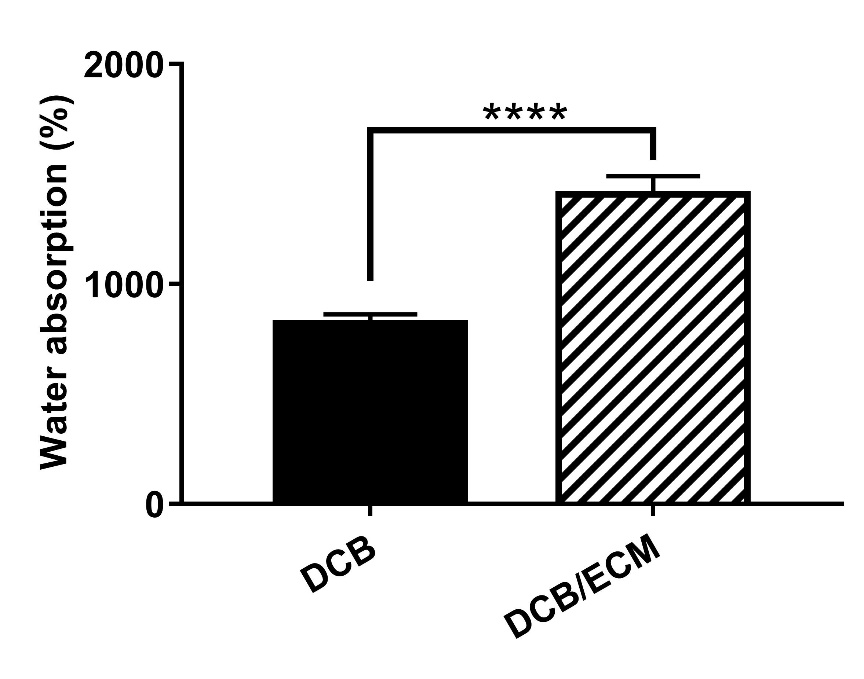
**

**Figure S1.** Water absorption of the DCB and DCB/ECM scaffolds. The values are presented as the means ± SD (n=5).

**
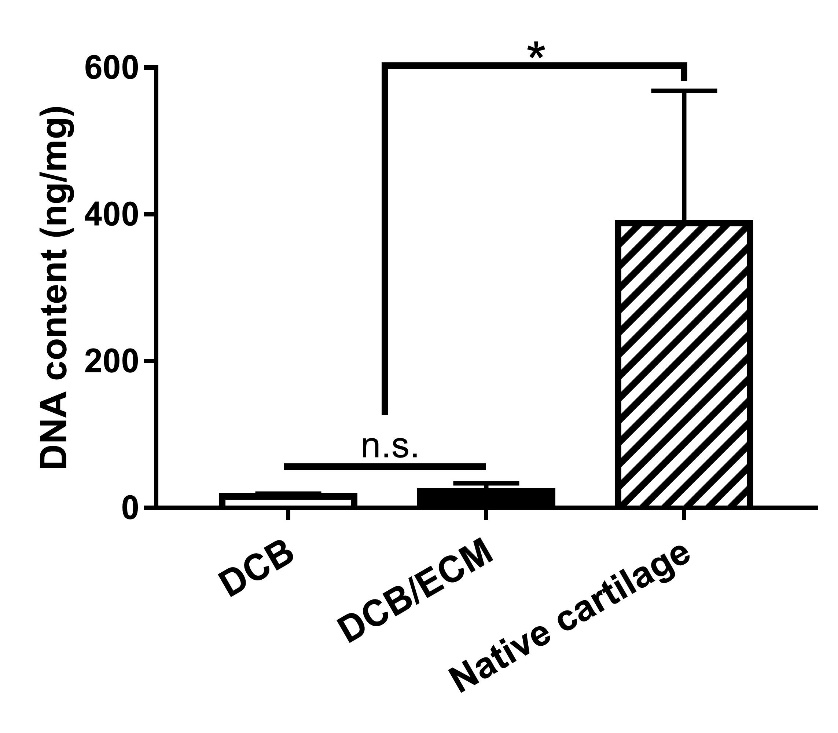
**

**Figure S2.** Residual DNA quantification of the DCB and DCB/ECM scaffolds. The values are presented as the means ±SD (n=3).

**
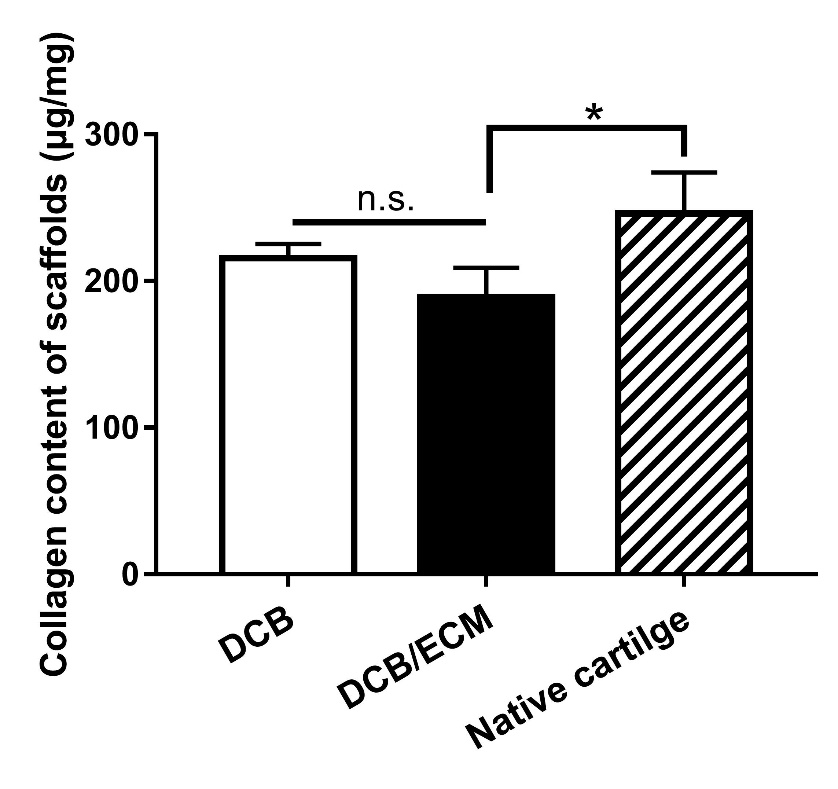
**

**Figure S3.** Total collagen content of the DCB and DCB/ECM scaffolds.

**
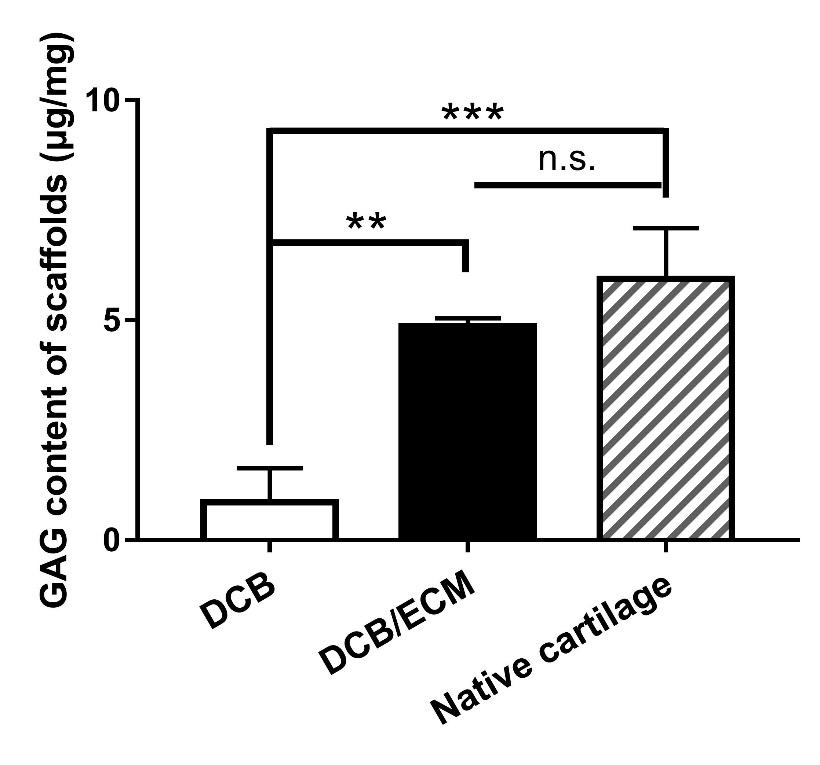
**

**Figure S4.** Total GAG content of the DCB and DCB/ECM scaffolds.

**
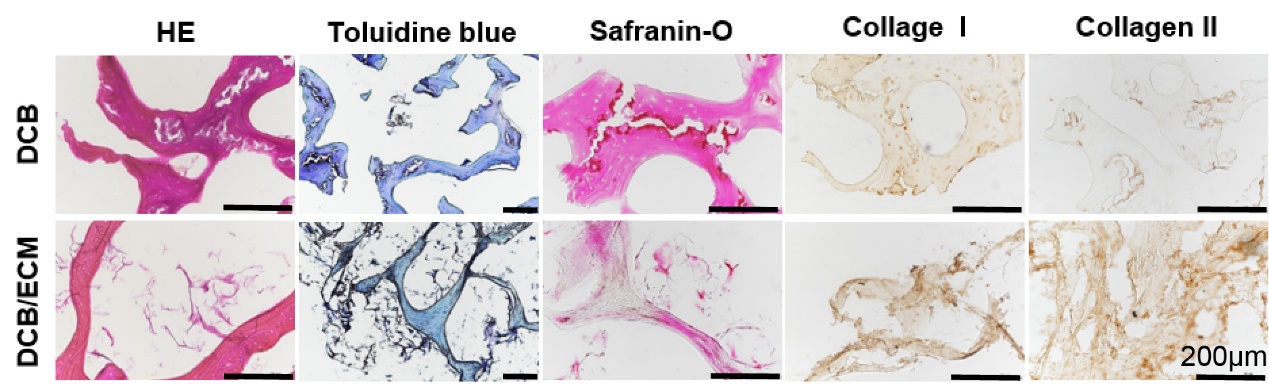
**

**Figure S5.** Histological analysis of the DCB and DCB/ECM scaffolds with H&E, Toluidine blue, Safranin-O, collagen I and collagen II.

**
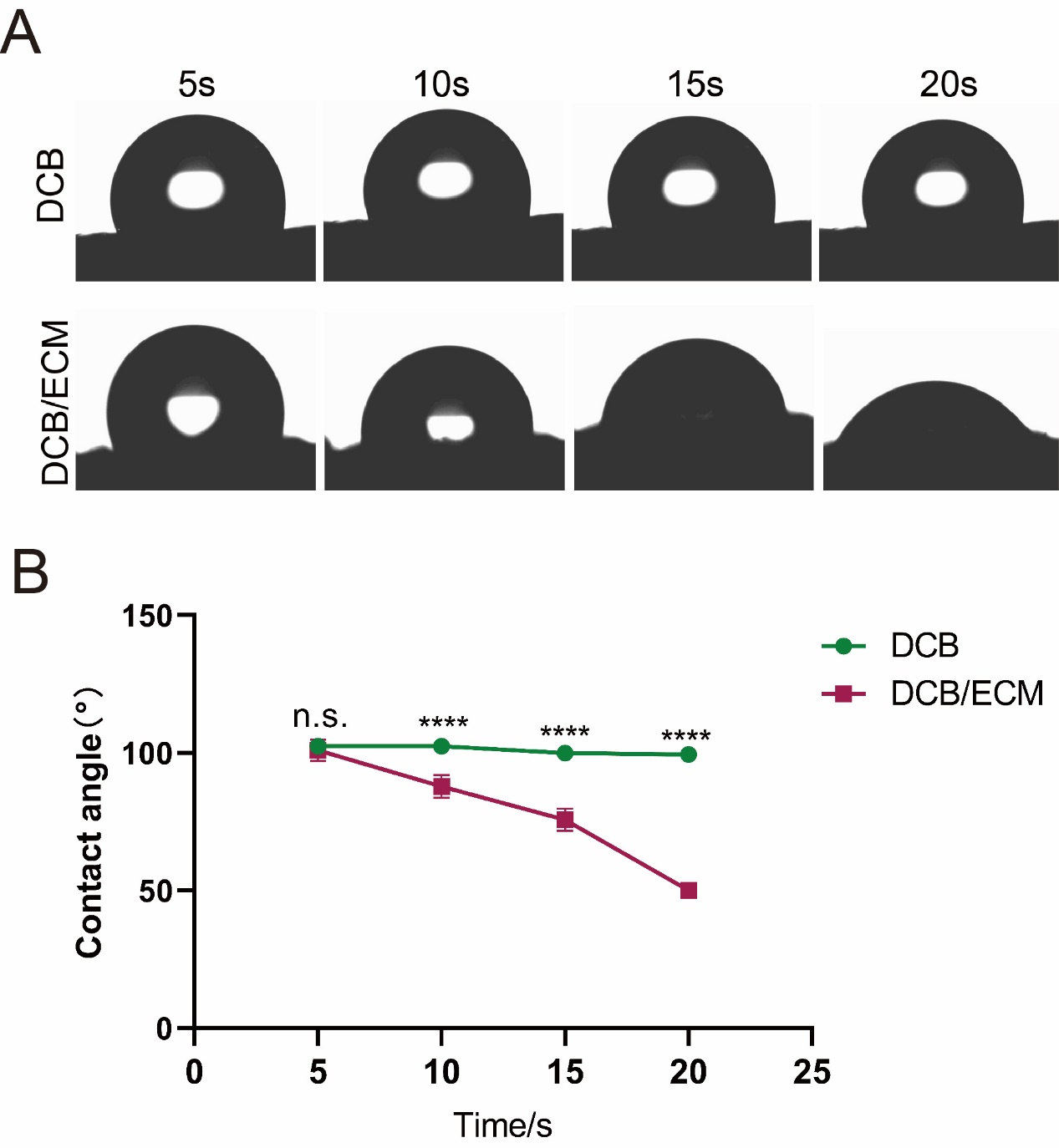
**

**Figure S6.** Contact angles of the DCB and DCB/ECM scaffolds. Four time points (5 s, 10 s, 15 s, and 20 s) were chosen. The values are presented as the means ± SD (n=4).

**
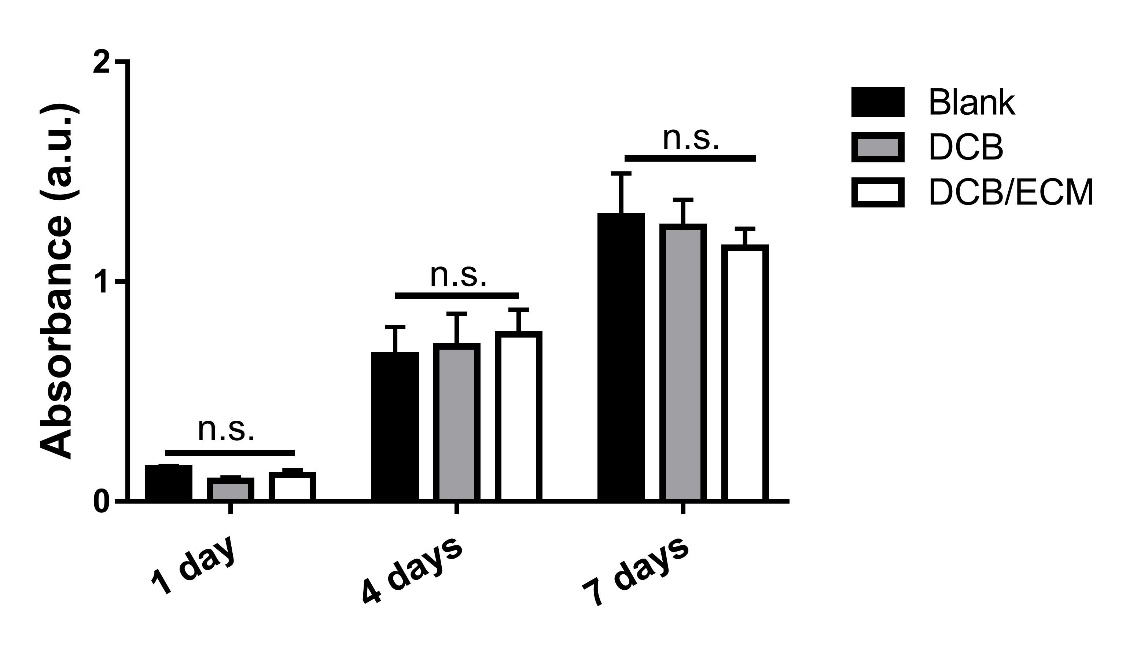
**

**Figure S7.** CCK-8 assay of IPFSCs seeded on DCB and DCB/ECM scaffolds for 1, 4 and 7 d. The values are presented as the means ± SD (n=5).

**
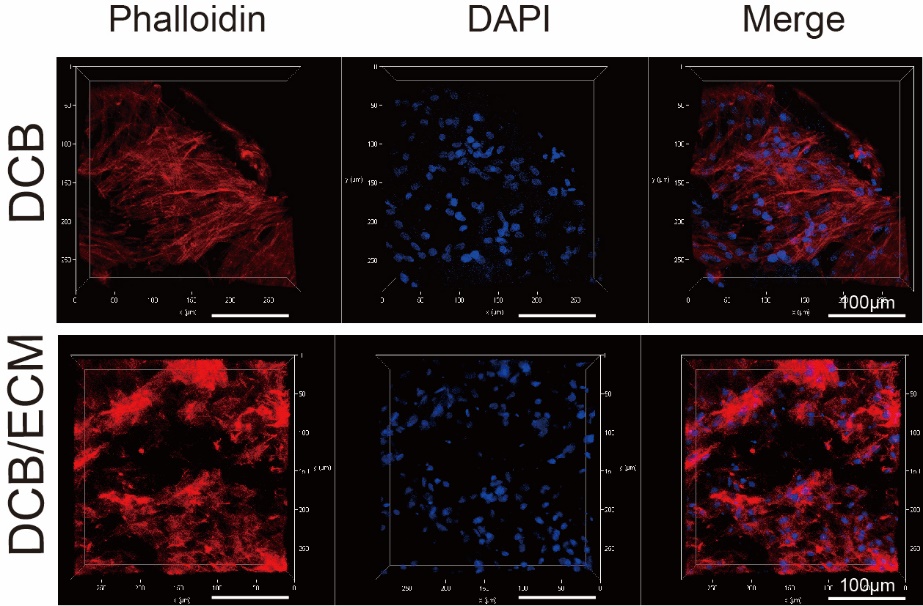
**

**Figure S8.** Phalloidin and DAPI staining analysis of IPFSCs cultured on DCB and DCB/ECM scaffolds for 7 d. The representative 3D reconstruction images show the cytoskeleton (red) and nucleus (blue).

**
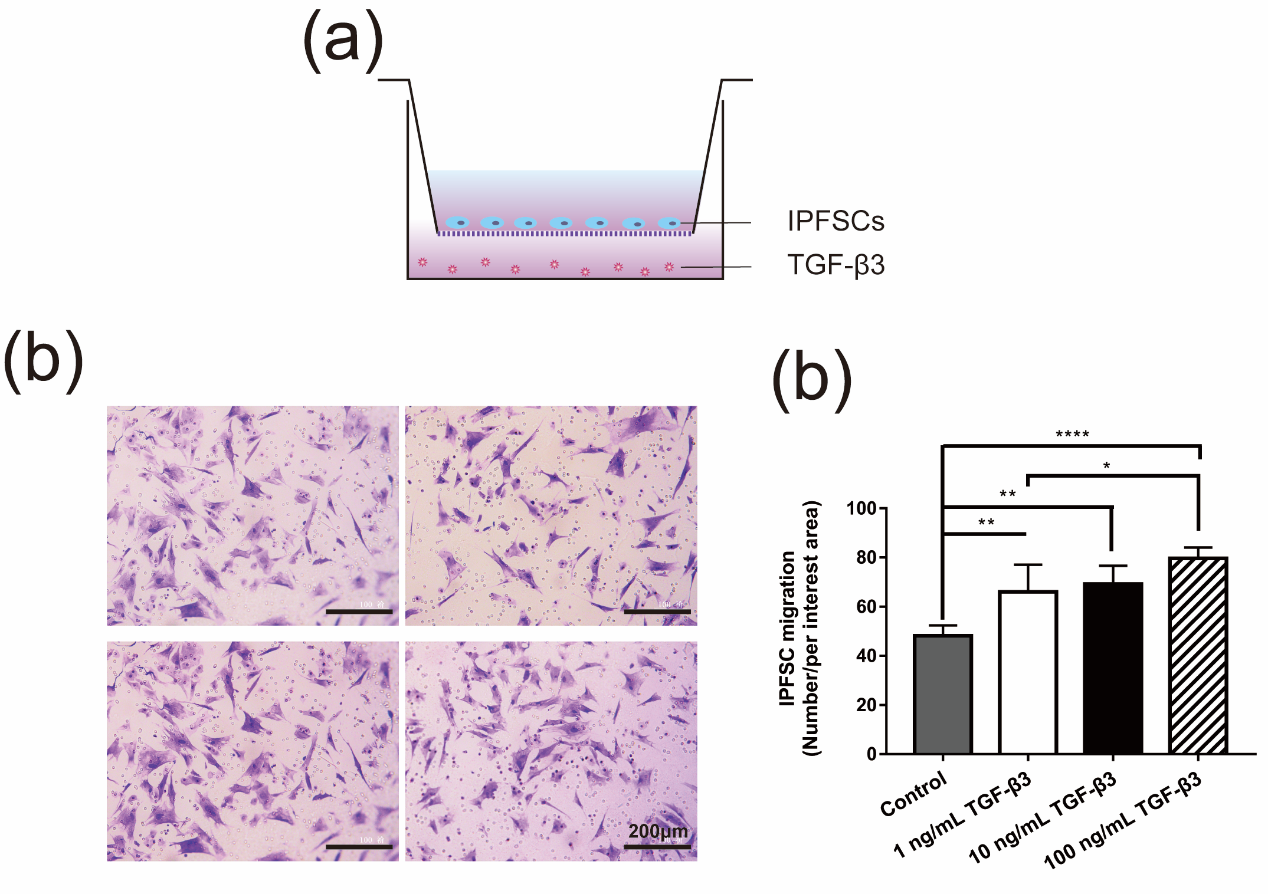
**

**Figure S9.** Effects of different concentrations of TGF-β3 on the migration of IPFSCs.
